# Role of Calcium and Low-Fat Dairy Foods in Weight-Loss Outcomes Revisited: Results from the Randomized Trial of Effects on Bone and Body Composition in Overweight/Obese Postmenopausal Women

**DOI:** 10.3390/nu11051157

**Published:** 2019-05-23

**Authors:** Jasminka Z. Ilich, Owen J. Kelly, Pei-Yang Liu, Hyehyung Shin, Youjin Kim, Yichih Chi, Kandauda K. A. S. Wickrama, Irena Colic-Baric

**Affiliations:** 1 Institute for Successful Longevity, Florida State University, Tallahassee, FL 32306, USA; 2 Nutrition Food & Exercise Sciences, Florida State University, Tallahassee, FL 32306, USA; drojkelly@gmail.com (O.J.K.); yichih.chi@gmail.com (Y.C.); 3 School of Nutrition and Dietetics, University of Akron, Akron, OH 44325-6103, USA; liu4@uakron.edu; 4 Retirement Research Institute, Samsung Life Insurance, Seoul 06620, South Korea; hyehyung@gmail.com; 5 General Nutrition Centers, Inc., Pittsburg, PA 15222, USA; youjinkim@gmail.com; 6 Human Development and Family Science, University of Georgia, Athens, GA 30602, USA; wickrama@uga.edu; 7 Faculty of Food Technology and Biotechnology, University of Zagreb, Zagreb 71000, Croatia; icolic@pbf.hr

**Keywords:** calcium and vitamin D supplements, low-fat dairy foods, weight loss, bone, body composition, body fat, muscle mass

## Abstract

Several studies have investigated the possibility of dairy foods and calcium (Ca) mediating weight and body composition, but a consensus has not been reached. We aimed to investigate weight-loss-related outcomes during intervention with low-fat dairy foods or Ca + vitamin D supplements, both as complements to hypocaloric diets. Overweight/obese Caucasian, early-postmenopausal women (*n* = 135) were recruited for a 6 month energy-restricted weight loss study complemented with either low-fat dairy foods (D; 4–5 servings/day), or Ca + vitamin D supplements (S); both to amount a total of ~1500 mg/day and 600 IU/day of Ca and vitamin D, respectively, or placebo pills (C). Bone mineral density (BMD) and lean and fat tissue were measured by Lunar iDXA. Serum and urinary markers of bone turnover were analyzed. Diet and physical activity were assessed with 3-day records. Participants on average lost ~4%, ~3%, and ~2% of body weight, fat, and lean tissue, respectively. The significantly better outcomes were noticed in participants in the D group regarding body composition (fat loss/lean tissue preservation) and in participants in the S group regarding the BMD outcomes, compared to those in the C group. Therefore, increasing low-fat dairy foods to 4–5 servings/day and/or increasing Ca & vitamin D intake by supplements (in those who are at the borderline dietary intake) may be beneficial for weight loss/maintenance and may lead to more favorable bone and body composition outcomes in postmenopausal women during moderate weight loss.

## 1. Introduction

At particularly high risk of becoming overweight are women in the early postmenopausal years, due to a combination of factors, including cessation of estrogen, decreased activity, and decreased resting metabolic rate [1,2]. Overweight/obese individuals often attempt to lose weight either on their own or as part of some organized programs to alleviate hypertension, cardiovascular disease, diabetes, or osteoarthritis. Although weight loss might improve these chronic conditions, it often leads to loss of bone [3,4,5,6,7,8,9] and muscle [10], especially without added resistance training [11,12]; the extent of the latter is dependent on many factors [13]. Decreased bone and muscle mass with weight loss may increase the risks for osteosarcopenia and subsequent fractures [8,14]. There is an obvious unfavorable effect on both bone and muscle with mechanical unloading during and after weight loss, as well as with the reduced protein and calcium intake (and reduced absorption of the latter) with the restricted energy consumption [15]. Other hormonal and cytokine secretion changes affecting bone and muscle during weight loss are addressed in the Discussion.

Another important aspect that needs to be addressed in aging population, especially in women, is that aging itself leads to reduction in bone mass/quality (osteopenia/osteoporosis) and muscle mass and strength (sarcopenia/dynapenia) and increase in, or redistribution of, body fat [2]. The coexisting presence of osteopenia/osteoporosis, sarcopenia and increased adiposity (either as overt overweight or fat redistribution or infiltration into bone and muscle) may result in a condition recently identified as osteosarcopenic obesity syndrome [16,17]. OSO has been associated with metabolic derangements, lower functionality, and poor diet, as reviewed recently [18]. Overall, contrary to previous beliefs, obesity may not be protective against osteoporosis and sarcopenia after a certain point, especially in older women [19,20,21], while weight loss may worsen each of the conditions.

Various foods and nutrients have been implicated to improve or worsen body weight and/or body fat accretion. Besides the energy and fat/carbohydrate intake, of the frequently studied foods and nutrients, dairy foods and calcium (Ca) have received considerable attention, yet their roles have not been elucidated. Various research groups agreed that Ca (with or without vitamin D) improved weight and body fat outcomes in overweight/obese individuals [22,23,24,25,26,27]. Additionally, some studies showed that intervention with dairy foods have resulted in better weight loss or weight management than could be attributed to their Ca content [28] and performed better than Ca supplements [26]. Higher low-fat dairy intake helped in weight loss efforts [29,30,31,32] and reduced waist circumference [32,33,34,35] and the percentage of body fat [29,31,33,34,36,37] in overweight and obese individuals. However, there is also a body of evidence, either from original studies of various durations and designs, or meta-analyses, showing no role of dairy foods or Ca in weight reduction and/or body composition improvements [38,39,40,41,42,43,44,45].

Therefore, our objective was to conduct a weight loss study and investigate whether weight loss and/or improvement in body composition, as well as the preservation of bone mass could be achieved easier with Ca + vitamin D supplements or low-fat dairy foods as complements to hypocaloric diets. We opted for a 6-month intervention study, a period long enough to detect changes in bone and body composition measured by dual energy x-ray absorptiometry (DXA), but not too long to cause diminished compliance and increased attrition rate among participants. We hypothesized that the hypocaloric diets with increased low-fat dairy foods or with Ca + vitamin D supplements, will be associated with greater weight reduction toward a healthier body composition and preservation of bone mass, in comparison to the control group (only hypocaloric diet), with the best outcomes in the low-fat dairy group. 

## 2. Methods

### 2.1. Overview of Design

This was a randomized, placebo-controlled clinical trial of three weight reduction protocols in overweight and obese Caucasian early postmenopausal women. The protocol involved moderate energy restriction (~85% of energy needs) for all participants divided in three groups: 1) Dairy group (D; including 4–5 servings/day of low-fat dairy foods); 2) Supplement group (S; with pills containing 630 mg Ca+400 IU of vitamin D/day); and 3) Control group (C; with placebo pills). After the baseline screening, participants were randomly assigned to one of the three groups, following a simple randomization procedure. The codes A, B, and D were assigned indicating two pill groups (supplement and placebo—A, B) and dairy group (D) and connected with the computer-generated random numbers for each participant. Further, A and B codes were double blinded and the allocator at Bayer, HealthCare LLC (pill manufacturer) concealed the codes until the end of the study. A dietitian reviewed participants’ diets to learn about each participant’s eating patterns and food preferences and subsequently design appropriate meal plan. Therefore, each woman received a tailored menu plan, which included her own food preferences. The intervention also included behavioral modification (with all participants) to help with dietary compliance and achieve possible long-term behavioral changes. 

#### 2.1.1. Weight Reduction/Management and Lifestyle Change Intervention

All participants went through a run-in period lasting 3 months, when they were seen every 2 weeks and given advice about their food choices and behavioral modification principles. During the run-in period, participants attended the biweekly 2-hour group sessions, with 5–8 members (of the same intervention group) and the multimodal cognitive-behavioral intervention, as well as nutritional education, as described previously [46]. This included standard behavioral, weight-management techniques (e.g., self-monitoring, self-efficacy, goal setting, stimulus control, etc.), as well as cognitive-behavioral techniques for restructuring and attitude change. The nutritional and behavioral modification sessions, conducted by dietitian and behavioral psychologist, respectively, were complemented with educational material, including lecture outlines, brochures, and practical tips on how to comply with the meal plan, focusing on maintenance of lifestyle change and relapse prevention [47,48]. 

#### 2.1.2. Dietary Plan

Meal plans were self-selected (based on self-preference) from the suitable and appropriate exchange lists based on the Dietary Guidelines for Americans [49]. The energy restricted diet was prescribed individually for each participant and was ~85% of energy needs for that individual, amounting to an overall energy deficit of 500–1000 kcal/day in order to elicit a weight loss of 1–2 lb/week. The Miffin–St Jeor equation [50] was used to calculate each participant’s maintenance energy needs. Afterwards, ~500 kcal was subtracted from the total energy need to elicit the desired weight loss. 

Participants in the D group were instructed to consume low- or reduced-fat dairy foods (4–5 servings/day) such as cheeses, skim or 1% milk, yogurt, pudding, low-fat ice cream and frozen yogurt. It was estimated that total diet provided ~1500 mg of Ca/day and ~600 IU (10 μg) of vitamin D. Participants in the S group received Ca citrate + vitamin D tablets (adjusted individually to provide the total of 1500 mg Ca/day and ~600 IU of vitamin D) and the C group received placebo (Bayer, HealthCare LLC, Morristown, NJ). The participants in the latter two groups were instructed to consume no more than two servings of dairy food/day. The compliance with Ca supplements/placebo was monitored by pill count, while that of dairy food intake by dietary records. In all cases, 24-h urine (see below) was analyzed for Ca excretion and bone resorption markers.

### 2.2. Participants and Inclusion/Exclusion Criteria

The inclusion/exclusion criteria have been described previously [20,21,46]. Briefly, the selected participants were generally healthy, 2–10 years postmenopausal, able to live independently and free of chronic diseases including severe osteoporosis (T-score ≥ −2.5 at any skeletal site) or fractures in the last six months, insulin-dependent diabetes, uncontrolled hypertension, liver disease, kidney disease/stones, cardiovascular condition, cancer, lactose intolerance, presence of any gastrointestinal disease, eating disorders, food allergies, or any other condition precluding them from participating in diet restriction and loss of weight. They could not be taking medications known to affect bone metabolism (such as hormone replacement therapy or corticosteroids) or metabolic rate and weight for the past 3 months. Further exclusion criteria included: current smoking (>1 pack of cigarettes/day) and excessive alcohol or caffeine consumption (>2–3 drinks/day), excessive exercise (although moderate exercise was allowed and encouraged), excessive body weight change or consumption of medically prescribed diets during the past 3 months. Only participants with total dietary Ca intake of ≤800 mg Ca/day were enrolled. If the qualifying participant was taking Ca supplements, she was asked to stop and was allowed 1 month of equilibration before enrolling in the study. Other mineral and vitamin supplements were allowed but were carefully accounted for (see Diet and Physical Activity Assessment). The study protocol was approved by the Institutional Review Board at the Florida State University. Written informed consent was obtained before participation in the study from each participant. 

### 2.3. Physical Measurements 

#### Anthropometry and Body Composition

Weight and height were measured in normal indoor clothing without shoes using a digital scale (Seca Corp., Model 707, Columbia, MD) and a wall-mounted stadiometer (Medart, St. Louis, MO), respectively, every 1–2 weeks in the run-in period, and then at 3 and 6 months, but only the measurements at baseline and six months are presented here. Waist and hip circumferences were measured to a nearest 0.1 cm with a plastic measuring tape (Issaquah, WA, USA) as the participant exhaled while standing straight. Waist circumference was measured at the smallest portion of the midsection, and hip at the maximum circumference around the buttocks on a horizontal level plain parallel to floor. The coefficient of variation (CV) for each measure was determined by measuring 10 participants at three separate times with the resulting CV% for weight, height, hip and waist circumference of <0.1%, 0.3%, 0.5%, and 0.5%, respectively, as described previously [20,21,46].

Body composition measurements were performed by dual energy x-ray absorptiometry using the iDXA instrument (GE Medical Systems, Madison, WI) with Encore 2006 software (version 13.11.016), as described previously [20]. The total body scan yields the analysis of the total body lean and fat mass and areal bone mineral density (BMD, g/cm^2^). The iDXA has a wider scan field and higher precision that can accommodate individuals up to 181 kg, avoiding the errors typically encountered when measuring overweight/obese individuals, and therefore providing a more accurate assessment of body composition [51]. The lean mass component refers to the total body mass, after excluding bone and fat mass and is usually used as a proxy for muscle mass. BMD of isolated skeletal sites, including spine (L2–L4), both left and right femurs (neck, Ward’s triangle, trochanter and shaft), and both forearms (ulna and radius at ultradistal and 1/3 site) were analyzed by specialized software for each region. During the measurements, the participants were in indoor clothing without any metal parts (including zippers) and were advised not to drink coffee or other diuretic drinks 3 h prior to the measurements [20].

The quality assurance of the densitometer was performed weekly by measuring the aluminum spine phantom (for bone mineral density, BMD) with the resulting CV of 0.45% for the entire study period. The measurements did not show drift during the study period, with an average BMD of 1.248 ± 0.01 g/cm^2^, ranging from 1.241–1.263 g/cm^2^. The %CV for different skeletal sites in-vivo was calculated from the repeated measurements (3 times each) on 10 overweight/obese women, comparable to the study participants. The %CV for the BMD of various skeletal sites was as follows: lumbar spine (L2–L4), 1.3%; femoral neck, 0.6%; whole femur, 0.7%; forearm (radius and ulna pooled together at 1/3 distance from styloid process) 0.9%. The CV% from the total body scans were: BMD (g/cm^2^), fat (kg), fat (%) and lean mass (kg) 0.6%, 0.8%, 0.8%, and 0.7%, respectively.

### 2.4. Clinical Chemistry

#### 2.4.1. Blood

Overnight fasting blood samples were obtained at the baseline and 6 months screening. Serum was separated from red blood cells and analyzed for routine tests, including serum Ca, protein, and creatinine, by the contracting laboratory (Quest Diagnostics, San Capistrano, CA), as described previously [52]. Remaining serum samples were stored at −80 °C and later analyzed for 25 hydroxy vitamin D (25(OH)D) (Immunodiagnostic Systems, Fountain Hills, AZ, sensitivity 5 nmol/l, intra-assay precision 5.9%, inter-assay precision 6.6%); intact PTH (Immunotopics, San Clemente, CA, sensitivity 0.6 pg/mL, intra-assay precision 4.7%, inter-assay precision 5.6%). Additionally, osteocalcin (Nordic Bioscience Diagnostic, Herlev, Denmark) and serum amino- (N-) terminal cross-linking telopeptides of type I collagen (NTx) (Wampole Laboratories; Princeton, NJ) were analyzed using commercially available enzyme-linked immunosorbent assay (ELISA) kits. All reagents were prepared and steps performed according to the manufacturer’s instruction with coefficients of variations < 10%. Each sample was tested in duplicate.

#### 2.4.2. Urine

Each participant received written instructions and was individually trained on how to collect a 24-hour urine sample, as described previously [53,54]. The compliance was self-reported; however, urinary creatinine was also used to screen for possible errors and incomplete collections (daily urinary output of creatinine is commonly used to estimate the adequacy of 24-hour collections of urine) [53]. The 24-hour urine volume was measured and the sample was analyzed by a routine chemstrip analysis. Thereafter, the sample was divided in aliquots and routinely analyzed for calcium, sodium, phosphorus, magnesium, and creatinine (Quest Diagnostics, San Capistrano, CA, USA). Urine carboxyl- (C-) terminal cross-linking telopeptides of type I collagen (CTx) (marker of bone resorption) was analyzed by using immunoassay kits (Immunodiagnostic Systems, Fountain Hills, AZ), according to the manufacturer’s instructions and all samples were tested in duplicate.

### 2.5. Dietary and Physical Activity (PA) Assessment

The participants were instructed by a dietitian on how to complete detailed 3-day dietary record (2 week and 1 weekend day) upon entering the study. Subsequently, they completed the records on a biweekly basis in the first 3-month run-in period and then at the end of the study (6 months). The nutrient intake from the records was analyzed with Food Processor^®^ (ESHA Research, Salem, OR), and the mean daily intake was calculated, including energy and all other macro- and micronutrients. The intakes of vitamin and mineral supplements were carefully recorded and included in the total nutrient analysis. The participants who qualified for the study but were taking Ca supplements were asked to stop taking them and if they agreed, their enrollment was delayed for 1 month after which time they were randomly assigned in one of the three groups, as described previously [20,46,55].

Physical activity was assessed using interview format with a modified version of the Allied Dunbar National Fitness Survey [56,57,58]. Subjects were asked about participation in recreational and sport activities of at least 4 kcal/min (intensity likely to produce health benefit) as well as about performing heavy housework, home improvement, gardening, and walking (distances of at least 1 mile). Each activity was assessed for frequency and duration and expressed in hours/week based on the average of the previous 4 weeks. Total activity score was calculated as well [57] and presented as hours/week. The records were obtained upon entering the study and then every 3 months, except in the 3-month run-in period when the PA journals were kept and evaluated on a biweekly basis (same as dietary records). Participants were also instructed to document their activities over a 24-hour period (within 2 week- and 1 weekend day), including the number of hours of sleep, utilizing Bouchard et al. questionnaire [58], from which energy expenditure as metabolic equivalents (MET) were calculated. Both dietary and activity records were reviewed by a dietitian (research assistant) during consultations to check for accuracy and completeness.

### 2.6. Questionnaires

Underlying factors such as education level, marital status, current smoking status, perceived health status, and medication intake were collected using a self-administered questionnaire at data collection sessions, as described previously [52], and used as confounders.

### 2.7. Statistical Analyses

**Figure 1:**
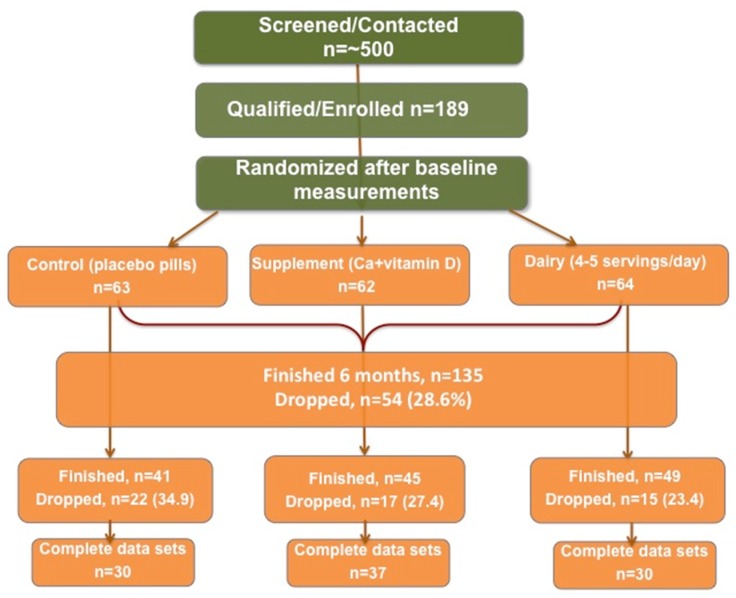
Flow chart of participants’ recruitment, randomization into groups, and attrition.

Most of the exploratory longitudinal analyses were performed via standard statistical methods such as repeated outcome measures using SAS and SPSS. P of ≤0.05 was considered significant. After checking for normal distribution of variables, the descriptive characteristics were calculated. Specifically, difference among three treatment groups (inter) or difference between baseline and 6 months within the same group (intra) were computed. Although, *n* = 189 subjects participated in the baseline measurements, there was an attrition of about 30% in 6-month follow-up. There were also missing data in some variables even among the subjects who completed the study as seen in Figure 1, presenting the participants’ recruitment, randomization into groups and attrition. This approach decreased the number of subjects/data points but was reflective of the actual data.

Additionally, although missing sample adjustments are widely criticized [59], we used Full Information Maximum Likelihood (FIML) to manage the missing values in order to utilize all the available data in the longitudinal repeated analysis models. The FIML offers substantial improvement over the traditional methods and research indicates that this approach is successful to manage up to 30% of missing data under the missing at random (MAR) and missing completely at random (MCAR) conditions enhancing the efficiency of analyses [59,60]. The missingness in the present data appears to be MAR. Specifically, testing a three factorial (Dairy (D) vs. Supplement (S) vs. Control (C) groups) design for continuous measures (weight, BMI, fat mass, lean mass, BMD, etc.) the repeated measures analysis of covariance (RM ANCOVA) was utilized. The analyses of interest were all variables whose interaction with time was assessed. Thus, the focus was on change, both overall and as a function of the three main factors (groups). Also, other demographic covariates (age, education), as well as nonhypothesized interactions of the three factors, and of each factor with selected covariates were examined. The statistically significant trends were obtained by calculating the difference between baseline and 6 months and regressing the change on each of the indicator variable (0 = Control, 1 = Supplement and 2 = Diary). In those models, the confounders were age, serum 25(OH)D, urinary Ca/creatinine, and total energy expenditure (MET) at the baseline.

Power analysis was calculated based on the results from the Chao et al. study examining the effects of weight loss on BMD of older women [6]. These pooled calculations resulted in an estimate of SD = 10.2 lbs. Hence, a medium effect size difference (SD = 0.5) between the respective weight losses of groups would be 5.1 lbs. Assuming standard Type I (α = 0.05), 2-tailed, and standard Type II (β = 0.20; power = 1 − β = 0.80) errors, this translated into requiring that an *n* = 44 is utilized for each of three groups for which to test the null hypotheses. Since covariates are of interest, an additional one subject per each df would be required for the repeated measures analysis of covariance, totaling *n* = (44 x 3) + 10 = 142 subjects. We recruited n=189 and ended with *n* = 135 at 6 months, Figure 1.

## 3. Results

A total of *n* = 189 women was recruited and *n* = 135 completed the 6-month follow-up (28.6% drop-out rate), as shown in flow-chart, Figure 1. Table 1 presents relevant characteristics of the continuing and noncontinuing participants at baseline. The participants’ measurements for BMD, serum, and urine lab values were all within the normal range. Other anthropometric and body composition measures show fulfillment with inclusion criteria (generally healthy, overweight/obese, early postmenopausal). No statistical difference was found in variables between continuing and noncontinuing participants.

Regarding the baseline characteristics of the continuing participants stratified in groups (not presented), although there was no difference in the age among the groups, the participants in the S group were longer in menopause (*p* < 0.05) compared to those in other two groups. The participants in the C group were significantly heavier and had higher BMI compared to those in the S group, but not to those in the D group. Likewise, total body fat (kg) was significantly higher in the C group participants, compared to the S group only, while there was no difference in the total % body fat among the groups. Other variables (as listed in Table 1) were not statistically significant among the groups at baseline.

Table 2 presents the baseline and 6-month BMD values of various skeletal sites, and bone and serum markers of the participants stratified by study groups with inter- (within the different groups at baseline) and intra- (within the same group at baseline and 6 months) differences. For these calculations, only the data from participants with complete data sets in each group were analyzed (see Figure 1).

At baseline, participants in C group had significantly higher BMD of each skeletal site (except lumbar spine and whole forearms) compared to those in the S group but not compared to the D group. On the contrary, CTx, the marker of bone resorption, was significantly higher in C and S groups’ participants, compared to those in the D group. There was no difference at baseline among the groups regarding the NTx, another bone resorption marker, and osteocalcin, typically referred to as the marker of bone formation (Table 2, baseline section). There was also no intergroup difference in serum 25(OH)D and PTH at baseline.

Table 3 presents dietary and physical activity variables at baseline and 6 months in the participants stratified by the study groups. At baseline, there was no significant intergroup difference in energy and macronutrient intake (including protein g/kg body weight). However, Ca intake was significantly lower in C groups’ participants compared to those in the D group, while vitamin D intake was the lowest in S groups’ participants compared to those in the other two groups. There was also no difference in physical activity variables among participants in different groups at baseline.

Regarding the 6 months results, as presented in Table 2 (6 months measurements section), there was no significant intragroup difference between baseline and 6 months in BMD, except for the forearms BMD in C group, which significantly decreased compared to baseline. Regarding bone markers, urinary CTx significantly increased in C group and decreased in S group, while there was no change in D group. Urinary Ca excretion, both total and normalized by creatinine (not presented) increased significantly only in S group’s participants, while potassium, magnesium, phosphorus and sodium (possible mediators of bone and/or muscle metabolism) remained unchanged. Serum PTH significantly dropped in S group’s participants only, while 25(OH)D significantly increased in both S and D groups’ participants compared to baseline levels. All other clinical parameters as health indicators (electrolytes, minerals) were within the normal ranges and remained unchanged.

Regarding dietary intake at 6-month assessment (Table 3, 6-months section), the intake of energy, carbohydrates, and fat significantly decreased (as expected) in each group compared to baseline (intragroup difference). Protein intake (both total and per kg body weight) in C and S groups’ participants also decreased compared to baseline, while that of D group’s participants slightly increased, although not significantly. Although energy and protein intake was slightly higher in D group’s participants compared to those in S and C groups at 6 months, it did not reach statistical significance among the groups. Ca intake significantly decreased in C group and increased in S and D groups’ participants, compared to baseline. Vitamin D was unchanged in in C and D groups’ participants, while it significantly increased in S group’s participants (as the result of supplements), compared to baseline. Other nutrients possibly affecting bone and/or muscle metabolism (potassium, magnesium, phosphorus, and sodium), decreased slightly in each group’s participants (not presented) as a result of lower energy intake. Magnesium and potassium were slightly below and sodium was above the recommendations in each group’s participants, both at baseline and after 6 months, while phosphorus was at the recommended level.

Regarding physical activity at 6 months assessment (Table 3), the total physical activity increased (mostly due to increased walking and/or recreational activities) in S and D groups’ participants compared to the baseline (intragroup difference). A similar situation was recorded for energy expenditure (MET).

The attendance to the group meetings was different among groups: participants in the S group had significantly higher attendance (4.7 ± 1.5) at group meetings than those in the D group (3.9 ± 1.8, *p* < 0.001), but not from those in the C group (4.3 ± 1.9). The compliance with placebo/supplements and low-fat dairy food consumption, assessed at 6-month point showed 82.7% and 73.0% compliance with placebo and Ca supplements in C and S groups, respectively, while participants in D group consumed an average of 4.6 servings of low-fat dairy foods.

**Figure 2:**
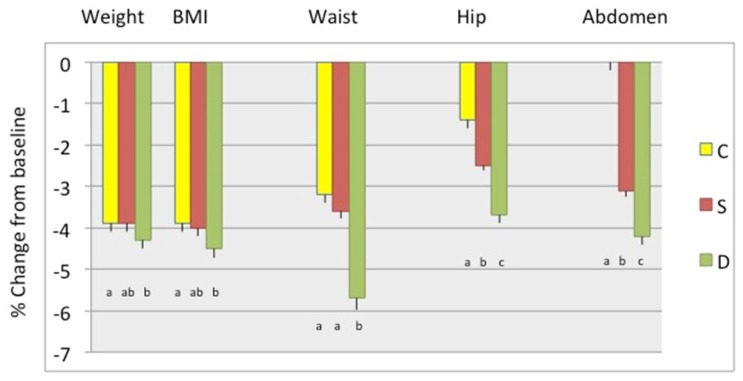
Percent change (mean ± SEM) in weight (kg), BMI (kg/m2), waist, hip, and abdominal circumference (cm), respectively, between baseline and 6 months among the groups. Only the complete data sets at baseline and 6-months were used. C-Control/placebo, *n* = 30; S-Supplement, *n* = 37; D-Dairy, *n* = 30. Bars with different letters indicate the statistically significant difference among the groups at 6 months.

**Figure 3:**
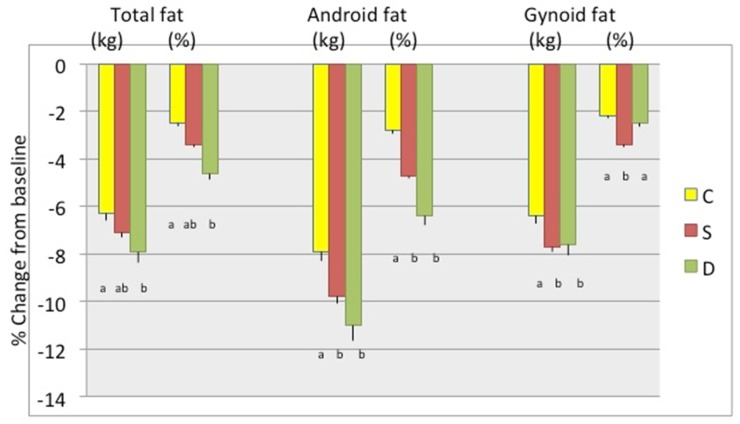
Percent change (mean ± SEM) in total body fat, android fat and gynoid fat (all kg and %), respectively, between baseline and 6 months among the groups. Only the complete data sets at baseline and 6 months were used: C-Control/placebo, *n* = 30; S-Supplement, *n* = 37; D-Dairy, *n* = 30. Bars with different letters indicate the statistically significant difference among the groups at 6 months.

**Figure 4:**
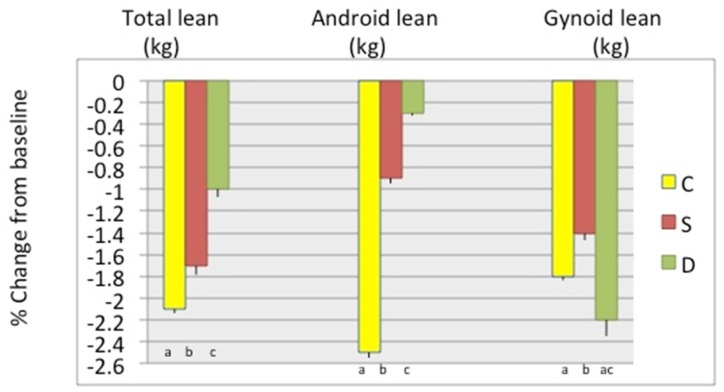
Percent change (mean ± SEM) in total body lean mass, android lean and gynoid lean (all kg), respectively, between baseline and 6-month among the groups. Only the complete data sets at baseline and 6 months were used: C-Control/placebo, *n* = 30; S-Supplement, *n* = 37; D-Dairy, *n* = 30. Bars with different letters indicate the statistically significant difference among the groups at 6 months.

**Figure 5:**
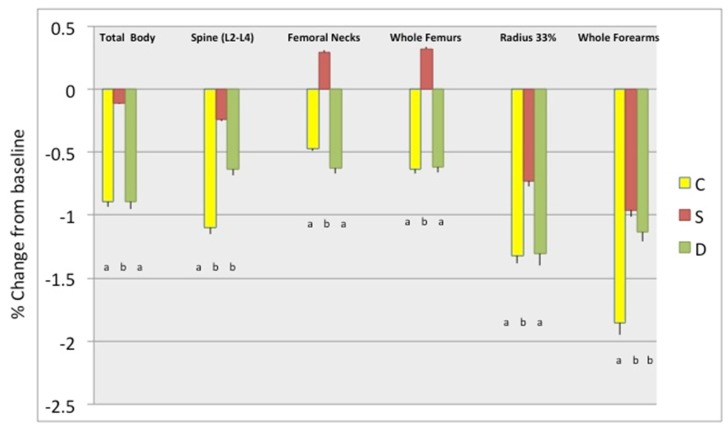
Percent change in bone mineral density (BMD) (mean ± SEM) of various skeletal sites between baseline and 6 months among the groups. Only the complete data sets at baseline and 6-months were used: C-Control/placebo, *n* = 30; S-Supplement, *n* = 37; D-Dairy, *n* = 30 were used for these analyses. BMD presents average values for both (left and right) femoral necks, both whole femurs (neck, Ward’s triangle, trochanter, and shaft), both radius 1/3 of styloid process, and both whole forearms (ulna and radius at ultradistal and 1/3 site). Bars with different letters indicate the statistically significant difference among the groups at 6 months.

Figure 2 presents weight and BMI, as well as waist, hip, and abdominal circumferences percent change between baseline and 6 months in participants stratified by study groups, showing the highest decrease in D group, followed by the S group, although the differences were not always statistically significant between the latter two groups. Figure 3 presents the percent change in total body fat (kg and %) and android and gynoid fat (kg and %). The change in total body and android fat was statistically higher in D and S groups’ participants compared to those in the C group, while S group participants had the highest loss in gynoid % fat. Figure 4 presents the percent change in total body lean, android, and gynoid lean tissue (kg). The difference in total body and android lean tissue loss was statistically lower in S and D groups’ participants compared to those in C group, while the loss of gynoid lean tissue was the lowest in S and highest in D group’s participants. Figure 5 presents the percent change in BMD of various skeletal sites over 6 months in participants stratified by study groups. The participants in S group had significantly lower loss in BMD of total body and radius compared to participants in C and D groups and significant gain in BMD of femoral neck and whole femur. Loss in spine and whole forearm was also significantly lower in S and D groups’ participants compared to those in C group.

Although the attrition rate was 28.6%, the missing data for some variables were even higher, up to 50% (e.g., dietary and physical activity variables, due to incomplete reports; serum and urine biomarkers, due to lack of serum/urine samples). Therefore, we conducted other statistical analyses, e.g., repeated measures ANOVA, MANOVA, autoregression models, etc. We present here the change-score-regression results in Table 4 and Table 5, where the D and S groups were tested in reference to C. Using the FIML for missing data and controlling for various confounders (biochemical, nutritional, physical activity, education), the analyses showed that intervention in the D group resulted in the highest decrease in total body fat, android and gynoid fat, and the lowest decrease in lean mass, with p values for two-tailed *t*-tests of about 0.6 and higher significance (<0.01) for one-tailed t-tests (Table 4). In similar analyses (Table 5), intervention in S group’s participants resulted in the significant outcomes regarding lumbar spine BMD and whole femurs (the lowest decrease). Table 4 and Table 5 show the final models for body composition and bone, respectively. These findings are in accordance with the separate tests performed and presented in Table 2 and Table 3 and Figure 3, Figure 4 and Figure 5, where values were controlled for age and BMI and the difference normalized for baseline values.

Regarding other lifestyle variables captured by questionnaires, only *n* = 5 (12.2%), *n* = 2 (4.4%) and *n* = 0 participants in the C, S, and D groups, respectively, smoked (1 pack of cigarettes/day or less). As far as the number of weight loss attempts in adult life (since high school), participants in C, S, and D had on average 1.8, 1.2, and 1.6 weight loss attempts (during which they lost ≥10 lbs), respectively. As far as the educational level, there were no differences (by Chi-square tests) in higher education levels among the groups, with over 50% and 20% participants completing college and/or graduate degrees, respectively. The rest of the participants completed either high school or some other vocational schools, equally spread among groups.

## 4. Discussion

Our data show that participants in both Ca + vitamin D supplements (S) group and low-fat dairy (D) group presented with better bone and body composition outcomes compared with participants in control (C) group after 6 months of moderate weight loss intervention. In general, the participants in S group tended to have better bone outcomes; lower decrease or slight increase in BMD in measured skeletal sites, while those in D group tended to have better body composition outcomes; higher decrease in fat and lower decrease in lean mass (Figure 2, Figure 3, Figure 4 and Figure 5). All participants consumed energy-restricted, but nutritionally balanced diets, and lost on average ~4% of body weight, ~3% of body fat, and ~2% of lean mass. Figure 2, Figure 3 and Figure 4 present changes in each group. The similar trends in findings were corroborated by the longitudinal, change-score-regression models, controlled for confounders (Table 4 and Table 5). In these models, a larger number of participants was used and the missing variables were manipulated by FIML.

The overall weight loss in our study was moderate and lower than what was achieved in some other studies with similar design. For example, in Zemel et al.’s study [61], the decrease in weight loss after 6-month intervention was 6.4%, 8.1%, and 10.9% for control, Ca supplemented, and dairy groups, respectively, among 32 obese adults. Our results have similar trends but a lower magnitude. Zemel et al. study did not examine the weight-loss effect on bones. Several studies in overweight/obese men and women revealed a BMD reduction during and after the weight loss [5,6,7,8,9,62,63]. This loss of a bone might be attenuated with Ca supplementation as reviewed earlier [64,65]. Jensen et al. 2001 observed a significant decrease in spine and total body BMD after 3 and 6 months of low energy diets in middle-age women. However, the spinal BMD loss was attenuated in the group that received 1000 mg/day of Ca supplements, prompting the authors to suggest that supplementation with Ca should be above the recommended levels during the weight reduction regimens [14]. In our study, Ca intake at baseline was below the recommendations in all participants, as per inclusion criteria. After 6 months, intake in C group’s participants dropped, but that in S and D groups’ participants increased, particularly in the S group. Thus, Ca and vitamin D intake in participants in the S and D groups was above or at the recommendations (DRI for Ca and vitamin D; 1200 mg/day and 15 μg (600 IU)/day, respectively [66]), probably not just preserving the bone but also causing a moderate BMD increase in some skeletal sites, and particularly so in comparison to BMD of C group’s participants.

In our study participants, markers of bone turnover, serum osteocalcin (formation) and NTx (resorption), remained unchanged. However, urinary CTx (also a resorption marker) significantly increased in the C group’s participants and decreased in the S group’s participants, indicating higher bone resorption in C and lower in S group’s participants (Table 2). While CTx and NTx are good indicators of changes in bone turnover during pharmacological interventions for osteoporosis [67,68], they may not change substantially in relatively healthy individuals, especially in the absence of fractures. In addition, there are large circadian variations in CTx and NTx making it harder to detect small changes, especially in urine [69]. This may explain why there was no change (or the change in CTx was too subtle to detect between groups) in our study population.

It is interesting to note that PTH decreased significantly only in the S group’s participants, while 25(OH)D significantly increased in participants of both the S and D groups after 6 months. Although the changes were small, and participants had normal values to begin with, they may indicate an improvement in bone metabolism, as well as compliance with the prescribed diets. Other studies have also shown a reduction in PTH from Ca supplementation [70,71]. Additionally, as shown earlier, maintaining lower PTH may also contribute to weight loss and prevent metabolic syndrome [72,73]. Increase in serum 25(OH)D, seen in our study participants, may partially be due to its release from the diminished body fat stores. Its increase in the S and D groups’ participants was statistically significantly higher compared to those in the C group. Since all participants had normal baseline levels of 25(OH)D, the clinical relevance of this change might not be that substantial. Decreases in serum PTH and increases in 25(OH)D have also been shown in other studies with Ca + vitamin D supplementation [74,75,76], although data are not consistent for dairy foods [77,78,79].

In the obesity epidemic era, numerous attempts for weight loss by millions of individuals became a health concern because of the bone and muscle loss during and/or after the weight loss. It is important to emphasize that bone, muscle and adipose tissues are interrelated organs, which are involved in secreting several biomarkers that can affect each tissue [17,80,81]. While loss of fat (particularly ectopic fat) could improve other conditions, including bone and muscle metabolism due to diminished secretion of some cytokines (e.g., interleukin-6 and tumor necrosis factor-alpha) both fat and muscle tissues, as components of weight, play important role in preserving bone mass via their loading effect. The lower mechanical force on bone and muscle during weight loss can raise the risk for fractures, particularly in older women [8,14]. Additionally, with the loss of body fat, the main source of extraglandular estrogen is reduced (of essence for postmenopausal women) [82,83,84,85], as well as the serum leptin which has dual effect on both bone and muscle. There is also an increase in serum cortisol and adiponectin, but a decrease in growth factors, all causing possible bone and muscle impairments [15]. In addition, the resorptive processes in bone (triggered by unloading) are shown to result in secretion of muscle-catabolic agents, possibly suppressing muscle strength and mass [86].

The reviews by Radak and Ilich discuss the studies of weight loss with Ca and/or dairy foods and their effects on bone [64,65]; however, the evidence to support an association of higher Ca intake and increased muscle mass or function is sparse [87] and, to our knowledge, no studies have been conducted to investigate the effects of Ca supplementation on muscle mass and/or strength. Calcium, being a bone antiresorptive agent, reduces bone-secretion of muscle-catabolic agents and may be indirectly sparing the muscle. Waning et al. showed in the metastatic mouse models that the bone breakdown results in higher muscle weakness due to elevated secretion of transforming growth factor (TGF)-beta from the bone surface, suppressing Ca^++^ induced muscle force production and strength [86]. The review [88] in burn patients with burn-induced muscle cachexia, who also suffer from bone loss, reported that early administration of bisphosphonates spares not just the bone, but the muscle as well. It is not quite known what the actual mechanism is, but the author suggests that the suppression of bone-resorptive agents’ secretion by bisphosphonates might exert the sparing effect on muscle mass and strength, similarly to that by calcium. Furthermore, meta-analyses of controlled trials reported a muscle sparing effect of dairy on muscle mass [89,90,91].

Overall, keeping in mind that adipose tissue is a major endocrine organ, secreting and/or metabolizing hormones (e.g., androgens), adipokines (e.g., leptin and adiponectin), and numerous cytokines, it affects both bone and muscle, often in a dual manner (positively and/or negatively) [2,17]. Therefore, any changes in adipose tissue might have an unanticipated influence on bone and muscle.

As proposed by Zemel [92,93] the mechanism of Ca mediating adiposity might be due to its intracellular level and its impact on lipogenesis and lipolysis in adipocytes. Consequently, low Ca intake results in lower serum ionized Ca^++^, stimulating PTH and calcitriol secretion, which both promote Ca influx into adipocytes. This increased intracellular Ca in adipocytes stimulates lipogenesis and suppresses lipolysis resulting in conservation of fat. High Ca intake (resulting in higher serum ionized Ca^++^) on the other hand, suppresses PTH and calcitriol response, stimulating lipolysis. This is also referred to as “calcium paradox”; paradoxical in that high cytosolic Ca caused by low dietary Ca will promote high lipogenesis and conservation of fat [94].

Regarding the mechanism of dairy foods possibly mediating weight/adiposity, besides Ca, several other components have been introduced. Another study by Zemel et al., comparing the effects of dairy foods and supplemental Ca on weight loss showed that women on reduced energy intake lost about 70% more weight on dairy foods and 26% more on Ca supplements (total Ca ~1200-1300 mg/day in both groups) compared to controls who had similar energy intake [61]. This effect could be due to either synergistic or separate influence of some of the bioactive components of dairy foods, including whey peptides [95], conjugated linoleic acid (although controversial [96]), and branched chain amino acids [97]. There are also some speculations of dairy foods influencing energy and adiposity balance, possibly through fat absorption, appetite or metabolic activity of gut microbiota [98]. Overall, nutrient-rich dairy-foods (rich in protein, some beneficial fatty acids, Ca, magnesium, potassium, vitamin D and some B vitamins), may be acting at multiple platforms and affecting several systems in the body. It was shown that particularly fermented dairy foods (like yogurts, cheeses) are associated with lower insulin resistance [99] and lower oxidative stress and inflammation [100], indicating their possible influence on obesity, type 2 diabetes and metabolic syndrome.

Despite the recent criticism of the role of dairy foods, particularly milk, in health, the available evidence supports the notion that adequate intake of dairy foods may protect against or improve many chronic conditions of modern time [101]. It is obvious that more investigation of the effects of Ca supplementation from different sources and amounts, as well as the dairy intake in weight reduction studies is necessary to elucidate the best treatment for preservation of bone and muscle and ultimately other chronic conditions caused by increased weight.

There are several limitations, as well as strengths to our study. This study was conducted in Caucasian overweight/obese early postmenopausal women (as per inclusion criteria) which limits the generalizability but increases the homogeneity of the study, particularly regarding the bone outcomes. Bone metabolism and BMD are different in Caucasian women compared to other ethnic groups (e.g., African Americans) and particularly to those of men. Therefore, the results of our study are limited to the population studied. It is important to keep in mind that DXA does not measure muscle directly, but the lean tissue which includes muscle and organs. During each weight loss, there is a loss of water, the highest occurring in the first couple of weeks. Since muscle contains much higher amount of water than body fat, it is likely that the proportion of water lost is higher from lean tissue. Over time however, the water loss stabilizes. During the 6-month duration of our study, it is likely that participants reached equilibrium in homeostatic regulation and the continued fat and lean mass loss occurred with a substantially smaller loss of water.

The extensive data collection (bone and body composition, blood and urine samples, dietary and physical activity assessments, and numerous lifestyle confounders) enabled the most comprehensive analyses and release of wide-ranging findings. Although we tried to limit the attrition rate and make the participation in the study as easy and pleasurable as possible (by conducting group meetings and counseling and having frequent contact with participants), the attrition rate still reached some 30%. Furthermore, the subsequent missing data-points were high (as seen typically in longitudinal intervention studies). We counteracted this by conducting the extensive analyses of the direct changes among the groups and differences between baseline and 6-month intervention, as well as the regression analyses using the FIML to offset the missing sample difficulties. Both types of analyses showed similar trends.

## 5. Conclusion

Our results show that hypocaloric diets with increased Ca + vitamin D intake and/or low-fat dairy foods during the 6-month intervention resulted in better weight and fat tissue loss, as well as in preservation of bone and lean tissue, compared to the hypocaloric diets only. The best outcomes were noticed in the participants in the low-fat dairy group, regarding the body composition outcomes and in those on Ca + vitamin D supplement group regarding the BMD outcomes. Therefore, increasing low-fat dairy foods to 4–5 servings per day and/or increasing Ca and vitamin D intake by supplements (in those who are at the borderline dietary intake) may be beneficial for weight loss/maintenance and may lead to more favorable bone and body composition outcomes. This is particularly important in view of the newly identified OSO syndrome [17], which simultaneously affects all three tissues and which has been associated with poor diet, poor lipid profile, reduced functionality, and increased frailty and risk of falls [18]. Therefore, the hypocaloric diet complemented with low-fat dairy foods or Ca + vitamin D supplements, in addition to causing a moderate weight loss, may delay or rescind OSO and any other related impairments in bone and body composition during the early postmenopausal years.

## Figures and Tables

**Table 1:** Anthropometrics, bone and body composition characteristics, and relevant lab values (mean ± SD) at baseline in continuing (*n* = 135) and noncontinuing (*n* = 54) participants.

Variable	Baseline Sample (*n* = 135)	Noncontinuing (*n* = 54)	
Age (y)	55.8 ± 4.3	55.9 ± 3.6	
Years Since Menopause (y)	6.1 ± 2.8	5.8 ± 2.6	
**Anthropometry/Body Composition**			
Weight (kg)	84.2 ± 14.0	82.4 ± 10.4	
Height (cm)	163.5 ± 5.5	162.6 ± 5.3	
Body Mass Index (kg/m^2^)	31.5 ± 5.1	30.7 ± 4.2	
Waist Circumference (cm)	97.2 ± 11.3	96.4 ± 10.2	
Abdominal Circumference (cm)	110.3 ± 12.3	108 ± 12.0	
Hip Circumference (cm)	117.1 ± 11.3	116.5 ± 10.8	
Total body fat (kg) Total body fat (%) Total body lean mass (kg)	39.3 ± 11.5 45.9 ± 6.8 42.7 ± 6.9	37.7 ± 8.6 45.4 ± 4.5 42.0 ± 5.0	
Android fat (kg) Android fat (%) Android lean (kg)	3.9 ± 1.4 55.5 ± 8.5 2.9 ± 0.5	3.7 ± 1.1 54.8 ± 7.6 2.8 ± 0.4	
Gynoid fat (kg) Gynoid fat (%) Gynoid lean (kg)	7.6 ± 2.1 55.4 ± 7.5 5.8 ± 1.0	7.5 ± 1.7 54.9 ± 4.8 5.7 ± 0.7	
**Bone Mineral Density (BMD) (g/cm^2^)**			**T-score**
Total Body Lumbar Spine (L2–L4) Femoral Necks * Whole Femurs * Radius 1/3 of Styloid Process * Whole Forearms *	1.137 ± 0.2 0.989 ± 0.2 0.933 ± 0.1 1.193 ± 0.2 0.685 ± 0.1 0.503 ± 0.1	1.133 ± 0.2 0.984 ± 0.2 0.930 ± 0.1 1.189 ± 0.2 0.681 ± 0.1 0.498 ± 0.1	-- −0.23 ± 1.4 −1.14 ± 1.2 −0.07 ± 1.3 −0.47 ± 1.2 --
**Lab Values**			**Normal range**
Serum PTH (pmol/L)	3.2 ± 1.7	3.1 ± 1.7	1.6–6.9
Serum 25(OH)D (nmol/L)	66.6 ± 27.9	66.3 ± 26.0	50–125
Serum Osteocalcin (ng/mL)	19.4 ± 7.1	19.5 ± 7.0	9–42
Serum NTx (nM BCE)	16.4 ± 4.4	16.0 ± 4.5	Elevated ≥26
Urine-CTx (ng/L)	13.8 ± 11.2	13.1 ± 11.0	Not established
Urine Ca/Creatinine (mg/24h)	135.4 ± 71.7	129.2 ± 51.8	30–275

* Average values for both (left and right) femoral necks, both whole femurs (neck, Ward’s triangle, trochanter and shaft), both radius 1/3 of styloid process, and both whole forearms (ulna and radius at ultradistal and 1/3 site). PTH, parathyroid hormone; 25(OH)D, 25 hydroxy vitamin D; NTx, serum amino- (N-) terminal cross-linking telopeptides of type I collagen; BCE, bone collagen equivalent; CTx, urine carboxyl- (C-) terminal cross-linking telopeptides of type I collagen; Urine Ca/Creatinine, urinary calcium normalized by urinary creatinine; There was no statistical difference in any of the variables between continuing and non-continuing participants (by 2-sample *t*-test, at *p* < 0.05)

**Table 2:** Bone mineral density (BMD) of various skeletal sites, markers of bone turnover and serum biomarkers in participants among the groups at baseline and after 6 months (mean ± SD); *n* = 30, *n* = 37, *n* = 30 for control/placebo (C), supplement (S) and dairy (D) group, respectively.

BMD (g/cm^2^) *	Baseline			*p* ^ab^	6 Months			*p*”
	C	S	D		C	S	D	
Total Body	1.159 ^a^ ± 0.11	1.098 ^b^ ± 0.10	1.123 ^a^ ± 0.09		1.154 ± 0.11	1.096 ± 0.10	1.112 ± 0.10	NS
Lumbar Spine (L2–L4)	1.201 ± 0.16	1.140 ± 0.14	1.174 ± 0.14	NS	1.176 ± 0.16	1.128 ± 0.15	1.156 ± 0.14	NS
Femoral Necks	0.962 ^a^ ± 0.13	0.888 ^b^ ± 0.09	0.919 ^ab^ ± 0.10		0.954 ± 0.12	0.896 ± 0.10	0.911 ± 0.10	NS
Whole Femurs	1.009 ^a^ ± 0.12	0.942 ^b^ ± 0.10	0.972 ^ab^ ± 0.10		1.004 ± 0.11	0.951 ± 0.11	0.968 ± 0.10	NS
Radius 1/3 of Styloid Process	0.700 ± 0.06	0.669 ± 0.08	0.672 ± 0.06	NS	0.695 ± 0.06	0.665 ± 0.08	0.651 ± 0.07	NS
Whole Forearms	0.512 ^a^ ± 0.05	0.485 ^b^ ± 0.05	0.494 ^ab^ ± 0.05		0.482^”^ ± 0.05	0.482 ± 0.06	0.480 ± 0.05	
**Markers of Bone Turnover**								
Serum Osteocalcin (ng/mL)	20.8 ± 7.2	20.2 ± 5.9	18.4 ± 6.2	NS	20.9 ± 8.4	19.7 ± 7.3	17.8 ± 7.0	NS
Serum NTx (nM BCE)	17.5 ± 3.9	16.5 ± 3.1	15.7 ± 4.8	NS	16.1 ± 4.7	15.0 ± 5.6	15.7 ± 4.6	NS
Urinary CTx	14.3 ^a^ ± 8.6	14.7 ^a^ ± 11.5	11.4 ^b^ ± 7.5		17.3^”^ ± 15.7	11.7^”^ ± 9.7	12.2 ± 10.6	
**Serum Markers**								
Serum PTH (pmol/L)	3.0 ± 1.4	3.1 ± 1.7	3.2 ± 1.8	NS	3.5 ± 1.3	2.8^”^ ± 1.2	3.0 ± 0.9	
Serum 25(OH)D (nmol/L)	65.8 ± 24.4	70.7 ± 32.7	66.6 ± 27.5	NS	71.9 ± 30.9	83.0^”^ ± 23.4	82.8^”^ ± 46.8	

Values are adjusted for age and body mass index (BMI); PTH, parathyroid hormone; 25(OH)D, 25 hydroxy vitamin D; NTx, serum amino- (N-) terminal cross-linking telopeptides of type I collagen; BCE, bone collagen equivalent; CTx, urine carboxyl- (C-) terminal cross-linking telopeptides of type I collagen; * Average values for both (left and right) femoral necks, both whole femurs (neck, Ward’s triangle, trochanter and shaft), both radius 1/3 of styloid process, and both whole forearms (ulna and radius at ultradistal and 1/3 site). p ^ab^ Statistically significant difference among the groups (intergroup). Values with different superscripts (a,b) are significantly different at baseline (2 sample t-test). p” Statistically significant difference within the group (intragroup). Values with different superscripts (“) are significantly different at 6 months compared to the baseline (paired *t*-test).

**Table 3:** Relevant dietary and physical activity variables (mean ± SD) at baseline and 6 months in participants stratified according to the study groups; *n* = 30, *n* = 37, *n* = 30 for control/placebo (C), supplement (S) and dairy (D) group, respectively.

Variable	Baseline			6 Months				
	C	S	D	p^ab^	C	S	D	p”
**Nutrients ^†^**								
Energy (kcal/day)	1744.4 ± 383.9	1781.0 ± 420.9	1782.2 ± 473.4	NS	1375.3^”^ ± 367.7	1367.7^”^ ± 340.2	1502.1^”^ ± 342.2	
Protein (g/day)	70.4 ± 16.9	76.0 ± 21.1	75.4 ± 22.0	NS	63.8^”^ ± 13.7	66.0^”^ ± 17.6	75.1 ± 23.8	
Protein (g/kg/day)	0.87 ± 1.7	0.93 ± 1.5	0.91 ± 1.6	NS	0.73^”^ ± 1.8	0.86 ± 1.6	0.98 ± 1.7	
Carb (g/day)	210.7 ± 55.2	208.0 ± 66.3	208.4 ± 59.5	NS	169.2^”^ ± 61.9	164.8^”^ ± 44.1	187.2 ± 44.7	
Fat (g/day)	67.3 ± 19.6	69.4 ± 20.0	69.3 ± 29.9	NS	48.6^”^ ± 17.1	49.3^”^ ± 22.6	50.8^”^ ± 24.3	
Total calcium (mg/day) *	863.9 ^a^ ± 323.6	910.5 ^ab^ ± 493.1	942.4 ^b^ ± 334.9		712.0^”^ ± 236.0	1672.3^”^ ± 494.6	1170.0^”^ ± 476.7	
Total vitamin D (IU/day) *	372.3 ^a^ ± 304.8	276.0 ^b^ ± 270.0	382.4 ^a^ ± 290.0		382.8 ± 454.2	808.6^”^ ± 401.0	375.8^”^ ± 255.1	
**Physical activity** **(hr/week) ^††^**								
Housework	1.3 ± 1.4	1.5 ± 1.6	1.5 ± 1.7	NS	1.6 ± 1.5	2.1 ± 1.7	1.6 ± 1.3	NS
Gardening	1.7 ± 2.8	1.4 ± 2.6	1.3 ± 1.9	NS	1.0 ± 1.2	1.3 ± 2.2	2.0 ± 3.0	NS
Do-it-yourself	0.2 ± 0.4	0.3 ± 0.7	0.2 ± 0.6	NS	0.1 ± 0.2	0.3 ± 0.7	0.4 ± 0.7	NS
Walking	2.4 ± 3.2	1.7 ± 2.7	1.9 ± 3.3	NS	2.8 ± 2.9	3.0 ± 3.4^”^	3.4 ± 3.6^”^	
Recreation	3.1 ± 6.2	2.5 ± 3.4	3.1 ± 3.3	NS	2.2 ± 2.5	4.3 ± 5.7^”^	3.6 ± 4.3	NS
Total Activity	8.4 ± 8.5	7.3 ± 6.5	8.1 ± 5.5	NS	7.7 ± 4.4	11.0 ± 8.1^”^	11.1 ± 8.0^”^	
MET	44.0 ± 4.6	43.8 ± 5.3	44.6 ± 6.1	NS	43.4 ± 19.3	62.5 ± 18.9^”^	64.9 ± 21.3^”^	

* Includes the amount from food and from the multivitamin/mineral supplements taken at baseline. MET, Metabolic equivalent. ^†^ By ANOVA and subsequent t-tests; ^††^ By Wilcoxon Rank Sums test due to the violation of normality assumption. p^ab^ Statistically significant difference among the study groups (intergroup difference). Values with different superscripts (a,b) are significantly different at baseline (2 sample t-test). p” Statistically significant difference within the same group (intragroup difference). Values with different superscripts (“) are significantly different at 6 months compared to the baseline (paired *t*-test).

**Table 4:** Predicting change from baseline to 6 months using Dairy group (*n* = 49) compared to Control group (*n* = 41) for total fat, android fat and total lean mass and confounders: age, metabolic equivalent (MET), serum 25 hydroxy vitamin D ((25(OH)D), and urinary Ca/Creatinine at baseline.

Total Fat	Stand Beta	*t*	*p*	Android Fat	Stand Beta	*t*	*p*	Total Lean	Stand Beta	*t*	*p*
Constant		0.197	0.845	Constant		0.56	0.955	Constant		−1.296	0.201
Age	−0.098	−0.777	0.441	Age	−0.080	−0.625	0.535	Age	0.129	1.001	0.322
MET	0.278	2.049	0.046	MET	0.272	1.979	0.053	MET	−0.169	−1.214	0.203
25(OH)D	−0.184	−1.430	0.159	25(OH)D	−0.173	−1.326	0.191	25(OH)D	0.297	2.249	0.029
Ca/Creat	−0.385	−2.869	0.060	Ca/Creat	−0.362	−2.671	0.010	Ca/Creat	0.174	1.270	0.210
**Dairy Gr**	−0.242	−1.923	0.060	**Dairy Gr**	−0.249	−1.956	0.056	**Dairy Gr**	0.243	1.889	0.065

**Table 5:** Predicting change from baseline to 6 months using Supplement group (*n* = 45) compared to Control group (*n* = 41) for total body and lumbar spine BMD and confounders: age, metabolic equivalent (MET), serum 25 hydroxy vitamin D and urinary Ca/Creatinine at baseline.

Femoral Neck BMD	Stand Beta	*t*	*p*	Whole femurs BMD	Stand Beta	*t*	*p*
Constant		−0.235	0.815	Constant		−0.020	0.984
Age	0.048	0.375	0.709	Age	−0.010	−0.082	0.935
MET	−0.110	−0.837	0.406	MET	−0.066	−0.511	0.611
25(OH)D	0.027	0.200	0.842	25(OH)D	−0.038	−0.294	0.770
Ca/Creatinine	0.219	1.555	0.125	Ca/Creat	0.296	2.158	0.035
**Supplement Gr**	0.276	1.973	0.058	**Supplement Gr**	0.234	1.946	0.063
